# Job stress and burnout among lecturers: a systematic literature review and meta analysis

**DOI:** 10.3389/fpsyg.2025.1673812

**Published:** 2025-11-20

**Authors:** Shi Yingying, Muhd Khaizer Omar, Normala Ismail

**Affiliations:** Department of Science and Technical Education, Faculty of Education Studies, Universiti Putra Malaysia, SerdangSelangor, Malaysia

**Keywords:** job stress, burnout, lecturers, meta-analysis, systematic literature review

## Abstract

**Background:**

The significance of higher education is essential for human progress. University lecturers face increasing demands in teaching, research and management, which often leads to increased job stress and burnout. Although numerous studies have investigated this relationship, the results are still inconsistent. This study performed an extensive literature review and meta-analysis to elucidate the association between lecturer job stress and burnout.

**Method:**

We conducted a systematic search in four major databases, SCOPUS, Web of Science, PubMed, and Google Scholar, and obtained all studies published until 2025. All studies set inclusion and exclusion criteria, and all cross-sectional studies were quality assessed using the JBI literature quality assessment form. Finally, 20 articles were included. The overall correlation coefficient of all literature was calculated by meta-analysis, and possible moderating factors such as country and gender were explored by subgroup analysis.

**Results:**

According to the meta-analysis, a strong positive correlation exists between job stress and burnout among university lecturers, as evidenced by a combined correlation coefficient of r = 0.452, a confidence interval of [0.380, 0.519], Z = 10.911, and *p* < 0.001, which was a moderate-high effect size. In the regression analysis, gender was not significant; however, in the subgroup analysis, cultural background and measurement tools exerted significant effects as moderator variables.

**Conclusion:**

This study aimed to further explore the relationship between job stress and burnout among university lecturers. The results provide comprehensive and accurate data support for how job stress affects burnout among lecturers in higher education, and have practical implications for universities to formulate targeted intervention measures.

**Systematic review registration:**

PROSPERO Registration: CRD420251073039, https://www.crd.york.ac.uk/PROSPERO/myprospero, https://www.crd.york.ac.uk/prospero/display_record.php?ID=CRD42023456789.

## Introduction

In 2019, the World Health Organization officially acknowledged burnout as a condition linked to workplace stress (World Health Organization, 2019). Occupational burnout was introduced in the 1970s as a psychological reaction to workplace interpersonal stress (Schaufeli et al., 2009). Historically, this notion has been extensively examined within professions that involve direct human services, including healthcare, education, psychotherapy, and social work. Moreover, burnout has also been conceptualized from a clinical or medical perspective. From a clinical perspective, burnout is classified as a work-related neuropsychological disorder and is listed in the International Classification of Diseases (ICD-10) (Schaufeli, 1998). In research on burnout, the definition proposed by Maslach (2018) is widely recognized and often used as a core reference. Burnout is described as a psychological condition characterized by emotional exhaustion, feelings of detachment or cynicism (known as depersonalization), and a reduced sense of personal accomplishment (as seen in professions involving interpersonal interaction). Emotional exhaustion is defined as extreme fatigue and exhaustion that an individual feels at work. This emotional exhaustion usually stems from long-term job stress and excessive workload (Inţă, 2021). Depersonalization describes a detached or dismissive attitude that individuals may develop toward colleagues or clients in the workplace. This is often manifested as a reduced sense of emotional identification with colleagues, clients, or students, similar to a state of “emotional numbness” (Sood, 2019). Personal accomplishment refers to the way individuals evaluate and validate their abilities and success in carrying out their professional responsibilities. Once emotional exhaustion and depersonalization intensify, personal accomplishment usually decreases, and individuals may feel lost and powerless because their work achievements are in urgent need of satisfaction (Alshurtan et al., 2024). One of the primary detrimental effects of burnout is a decrease in overall job performance (Batayneh et al., 2019). Burnout is correlated with reduced job satisfaction, lower organizational commitment, and an increased likelihood of resignation. The emotional weariness aspect of burnout is significantly associated with adverse personal consequences. Specifically, it is linked to health issues, diminished well-being, and multiple forms of substance misuse. Also, it can exacerbate an individual’s mental health, resulting in anxiety, despair, and diminished self-esteem (Patel et al., 2018).

Burnout is a widespread problem that can affect individuals in a variety of professions, such as students, journalists, athletes, judges, librarians, and even the unemployed (Burisch, 2006). In addition, research evidence shows that occupations that serve humanity, including healthcare providers, educators, and social service professionals, are particularly prone to experiencing burnout (Durr et al., 2014). Academic staff in higher education around the world face considerable stress and burnout, a phenomenon documented in multiple studies. Evidence suggests that burnout among educators is widespread across different countries and regions (Durr et al., 2014; Schaufeli et al., 2017). For instance, an online survey conducted by UNESCO IICBA among university lecturers in Kenya revealed that burnout symptoms, including emotional exhaustion, depersonalization, and reduced personal accomplishment, were relatively common within this population (UNESCO IICBA, 2022). Similarly, a survey by the American Psychological Association found that 64% of university faculty reported experiencing work-related burnout, with 19% indicating high levels and 15% reporting severe burnout (APA, 2023). In Europe and North America, further analyses suggest that nearly 30% of faculty members in higher education institutions experienced significant burnout during certain periods (Johnson et al., 2019). Moreover, in Southeast Asia, the mental health of educators was markedly affected by the COVID-19 pandemic, with burnout symptoms observed in up to 44% of participants (Abdul Aziz and Ong, 2024). The pandemic also caused widespread educational disruptions, affecting over 87% of students globally, which in turn placed unprecedented pressure on educators (UNESCO, 2022; Araújo et al., 2020). According to Karaköse et al. (2022), university teachers are among the most stressed occupational groups. However, Workplace stress is not a new phenomenon; it has long been associated with both mental and physical health problems. Burnout is a state caused by persistent stress, and teachers are particularly susceptible to chronic stress. Numerous studies have shown that teacher burnout has a negative impact on their self-efficacy, self-confidence, motivation, self-esteem, work efficiency, professional engagement, and overall job satisfaction (Skaalvik and Skaalvik, 2014; Herman et al., 2018). If burnout is not recognized and managed, educators may experience persistent anxiety, physical discomfort, and job resignation (Schaufeli et al., 2017).

The causes of burnout have been studied from both personal and organizational perspectives. There are some personal factors that contribute to anxiety (Almoied, 2023). Other researchers have argued that the causes of burnout are external, and that organizational and management systems play a greater role in why employees feel burned out (Freudenberger, 1977; Maslach and Leiter, 1997). Sillero and Zabalegui’s (2018) emphasized that the observed burnout was primarily related to nurses’ work environment and organizational factors, while personal factors and demographic characteristics were relatively minor. Job stress has a double-edged sword quality in the workplace. Moderate stress can be motivating, but excessive job stress can adversely impact employees, leading to heightened burnout and increased work-related complaints. Work-related factors are crucial in the development of burnout (Maslach and Leiter, 2016). In particular, various work characteristics such as excessive job demands, time stress, conflicting roles, confusion about roles, and insufficient support and autonomy are important triggers for burnout. Among them, the lack of a supportive environment and a sense of control over resources are also major drivers of anxiety and burnout (Wang et al., 2023; Sarwar et al., 2023). The potential causes of faculty burnout can be attributed to a complex interplay of factors. First, lecturers face a dual dilemma between promotion pressure and external expectations. Furthermore, a long-term lack of fulfillment in academic advancement, teaching reform, and social service is a significant factor (Zhou and Wang, 2025). These factors not only increase teachers’ anxiety, but also make their participation in teaching mechanical, neglect their scientific research responsibilities, and thus cause burnout.

### Burnout and job stress

Burnout is often conflated with stress. Although symptoms may share considerable similarities, it remains crucial to acknowledge the significant differences. While stress may intensify burnout, it is not the fundamental cause of this phenomenon (Burisch, 2006). Job stress is viewed as a response to immediate pressures and challenges in the workplace, while burnout is a deep, lasting emotional drain that accumulates when an individual is in a prolonged high-stress setting (Raymaker et al., 2020). Job stress is typically situational and short-term, while burnout represents a persistent state of drain that can significantly affect an individual’s overall quality of life and work performance (Raymaker et al., 2020; Parker and Tavella, 2022).

Burnout represents a mental illness arising from extended occupational stress (Thomas, 2004; Bianchi et al., 2015; Salvagioni et al., 2017). Burnout is often accompanied by boredom and negative attitudes towards work, causing individuals to feel ineffective and detached from work (Van Bakel et al., 2018; Hewitt et al., 2020). Its impact is not limited to the individual’s mental health, but also affects work performance, quality of life and interpersonal relationships. For example, the manifestations of burnout often include significant anxiety and depression symptoms (Bianchi and Schonfeld, 2021; Renaud and Lacroix, 2023). Definition of job stress. In contrast, job stress refers to the negative reactions felt by individuals at work, especially the psychological state that occurs when there is a significant imbalance between work requirements and personal coping ability (Gunasekra and Perera, 2023). Among them, this study also includes perceived stress as a type of job stress. Perceived stress describes how a person subjectively assesses and interprets the stressors in their environment. It involves an individual’s perception of work requirements, time constraints, and responsibilities, and the impact of these factors on their own psychological and physical health. For university teachers, perceived stress usually comes from teaching burden, scientific research requirements, student management, and work-life balance (Mache et al., 2016; Mohammed et al., 2019). Perceived stress is closely related to job stress, and the former is often a subjective reflection of the latter. An increase in job stress will lead to an increase in teachers’ perceived stress, and vice versa (Dėdelė et al., 2019; Mohammed et al., 2019). For example, when teachers face overloaded teaching and research tasks, they not only feel increased job stress but may also subjectively feel higher stress, which affects their mental health and work performance. Chen and Silverthorne (2008) characterized job stress as a form of work-related psychological stress, emphasizing employees’ capacity to manage specific workplace situations through their skills. Job stress can arise from various factors, including workload, role ambiguity, inadequate resources, and workplace conflict, potentially resulting in adverse emotional and physical outcomes (Batran, 2019; Prasad et al., 2021). Although job stress can occur independently of burnout, elevated levels of job stress serve as a key precursor, and prolonged exposure to stress significantly heightens the likelihood of developing burnout (Bianchi et al., 2015; Parker and Tavella, 2022).

Brewer and McMahan (2003) devoted himself to exploring the root causes of stress and burnout in the teacher group and pointed out that the core lies in the mismatch between individuals and the environment. He believed that stress and burnout arise from inconsistencies at multiple levels: including the objective reality of the work environment, the subjective perception of the environment by teachers, and the gap between job requirements and individual abilities or resources. Pandey and Tripathi (2001) identified role ambiguity and political pressure as the primary contributors to job burnout. Teaching, as a profession, is inherently stressful, with role ambiguity being one of the key challenges. According to Amimo (2012), every teacher may face burnout in their career. Once they fall into a state of burnout, many teachers often do not know how to deal with it and do not understand its root causes. This phenomenon is largely attributed to the increasingly complex and expanding structure of the education system. With the continuous evolution and increasing requirements of the education environment, teacher burnout has become a major and urgent issue in the current education field. Salami (2011) identified work pressure, social support, and individual personality traits as key contributors to job burnout. He categorized these influences into two overarching domains: environmental and personal factors. Shoaga et al. (2015) highlighted that excessive expectations and psychological strain were primary sources of occupational stress among educators, whereas feelings of exhaustion and discouragement at work were identified as the principal drivers of burnout. Among them, the important factor causing university teacher burnout is excessive workload. Because as colleges and universities continue to increase their requirements for scientific research output and teaching quality, teachers often face overloaded teaching and research tasks, which makes it difficult for teachers to balance between course preparation, teaching and scientific research projects, and the workload continues to increase, thus causing job stress. In addition, teachers also need to participate in administrative management, guiding students and other tasks, all of which may lead to emotional exhaustion (Pei et al., 2024). In addition, personal factors cannot be ignored. Teachers’ personality, ability to cope with stress, self-efficacy, etc., will affect their burnout level. Some studies have shown that lower self-efficacy and weaker adaptability may make teachers show a higher tendency to burnout when facing work pressure (Pu et al., 2017). In summary, teachers’ mental health, lack of family life and social support systems, personal psychological quality, self-efficacy and ability to cope with stress are also important factors leading to teachers’ professional burnout.

Research indicates that university lecturers experience elevated job stress and burnout (Li et al., 2023). However, further investigation is needed to explore the strong connection between these two factors among university lecturers. A study by Reddy and Poornima (2012) examined occupational stress and burnout among university teachers in India, revealing that 74% experienced medium to high levels of occupational stress, while 86% reported suffering from burnout. University-level educators experience significant pressure, resulting in organizational inefficiencies, elevated turnover rates, absenteeism, diminished work quality, increased healthcare costs, and lower employee job satisfaction. Stress occurs when lecturers perceive their work environment as depleting or surpassing their available resources, leading them to view it as a threat. Lecturers with elevated expectations and a strong drive to meet teaching objectives frequently experience higher levels of stress and burnout. Job stress is a key factor in predicting various dimensions of lecturer burnout, such as emotional exhaustion, depersonalization, and reduced sense of personal achievement (Yu et al., 2015).

### The current review

A large number of research literatures show that lecturers’ occupational stress and burnout are highly correlated. Although existing studies have analyzed job stress and burnout separately, in the current research literature, systematic literature reviews and meta-analysis on the relationship between lecturers’ job stress and burnout are still relatively scarce. Most existing studies focus on individual variables or only focus on meta-analysis of job stress or occupational burnout (Lee et al., 2011; Burman and Goswami, 2018; García-Carmona et al., 2019), and there are relatively few systematic discussions on the relationship between the two variables. Meanwhile, much of the relevant literature focuses on other occupational groups, such as teachers, police officers, nurses, and doctors (West et al., 2016; Queirós et al., 2020; Membrive-Jiménez et al., 2022). These studies primarily focus on high-stress work environments such as clinical or elementary education settings, while attention to higher education lecturers is relatively scarce. Even in meta-analyses focusing on teachers, the differences between elementary and higher education lecturers’ work often fail to distinguish between them. Compared to other occupational groups like nurses and police officers, lecturers face unique job stressors. While nurses and police officers experience stress primarily from high-risk work environments and emotional labor, lecturers, faced with teaching loads, research tasks, and administrative responsibilities, may experience different types of burnout mechanisms. Higher education lecturers’ work involves not only heavy classroom teaching but also extensive research and administrative responsibilities. This complex workload significantly differs from that of other occupations in terms of the risk and manifestations of burnout faced by lecturers. Therefore, this study focuses on the relationship between work stress and burnout among higher education lecturers through a systematic review and meta-analysis method, aiming to fill the research gap in this field and provide a more detailed occupational classification perspective for burnout research, especially to provide a theoretical basis for policy formulation and intervention measures for occupational health management in the context of higher education.

Systematic reviews offer a structured approach to gathering research evidence, aiming to reduce potential bias during the selection, evaluation, and integration of studies. Although meta-analyses are often a central feature of systematic reviews—serving as a quantitative method to combine results from multiple studies—it is feasible to carry out a systematic review without including one (McKenzie et al., 2016). Conversely, researchers can also perform a meta-analysis independently of a complete systematic review. Nevertheless, the most robust form of evidence is generally considered to be a systematic review that incorporates meta-analytic techniques (Rao, 2023).

Furthermore, variables including age, gender, and cultural background may influence the relationship between lecturers’ occupational stress and burnout. For example, the study by Zulkarnain et al. (2015) showed that there was a positive correlation between the length of service of female lecturers and the degree of burnout, that is, female lecturers with longer service were more likely to feel burnout, which was related to the workload and family roles they undertook. Portuguese lecturers experienced higher levels of burnout during the COVID-19 pandemic, and female teachers experienced higher levels of burnout than men, a phenomenon that was closely related to the country’s social support system and professional culture (Castro et al., 2023). In contrast, studies on Turkey showed that although gender differences had no significant effect on the burnout of young teachers, the lack of national policies and the increase in teachers’ workload were still important factors affecting burnout (Seis, 2023). Therefore, we have reason to suspect that this association may be different under these moderating factors.

In conclusion, this research aims to fill the existing vacuum by providing a comprehensive analysis of occupational stress and burnout among university professors. The findings aim to contribute valuable insights for improving mental health strategies within the academic profession and to guide future studies with a more structured and coherent research framework.

## Objective

This study not only quantifies the correlation strength between stress and burnout, but also further analyzes potential moderating factors, including cultural background, type of occupational burnout measurement tools, and sample gender ratio, to reveal whether these factors affect the size and direction of the effect size, thereby providing a theoretical basis and intervention suggestions for university administrators and education policy makers. Based on this, the specific research questions are as follows:

1. Clarify the overall correlation between job stress and occupational burnout in the lecturer group.
2. Explore the heterogeneity of this relationship under different research conditions.
3. Test whether a series of potential moderating variables (such as cultural background, burnout measurement tools, and sample gender ratio) affects the effect size.

### Meta-analysis questions

*RQ1*: How strong is the overall correlation between job stress and burnout in the lecturer group?

*RQ2*: Is there significant heterogeneity in the effect size between studies?

*RQ3*: Do different cultural backgrounds moderate the relationship between job stress and burnout?

*RQ4*: Do different burnout measurement tools affect the strength of the effect size?

*RQ5*: Does the gender composition of the sample have a moderating effect on the relationship between job stress and burnout?

## Methodology

Before we started searching the databases, we first searched for keywords and titles in PROSPERO to check whether there were similar literature reviews and meta-analyses. We then registered in PROSPERO (CRD420251073039). The procedures and reports of the studies were conducted according to the PRISMA checklist (Page et al., 2021).

### Search strategy

The search strategy combined subject terms (such as MeSH) and free terms, combined by Boolean logic operators (AND/OR), aiming to comprehensively cover cross-sectional research literature. The specific search strategy is as follows:

- Fields: Abstract, Keywords, Title
- Terms: ((“job stress” OR “occupational stress”) AND (“burnout” OR “job burnout” OR “occupational burnout”) AND (“lecturer*” OR “faculty” OR “university teacher*” OR “academic staff” OR “college instructor*”)).

### Inclusion and exclusion criteria

Initially, studies were required to include relevant keyword combinations based on the predefined search strategy. Furthermore, since this research integrates a meta-analytic component in addition to the systematic review, only quantitative studies reporting statistical associations between job stress and burnout were eligible. Lastly, all selected articles had to be written in English and published in peer-reviewed academic journals. The detailed eligibility criteria are outlined below.

#### Inclusion criteria

- Participants: Higher education lecturers, including university teachers, higher vocational teachers, college instructors, or academic faculty.
- Exposure and Outcome: Studies examining job stress, burnout, or the relationship between the two.
- Data: Sufficient statistical information for effect size computation
- Design: Quantitative studies
- Instruments: Use of validated tools
- Language: English.
- Type: Peer-reviewed journal articles.

#### Exclusion criteria

- Participants outside higher education.
- Qualitative studies, reviews, conference abstracts, editorials.
- Articles lacking extractable quantitative data.
- Non-English publications or gray literature.
- Theoretical papers without data.

### Data extraction

A pre-designed standardized form was used to extract the main content from each study: authors, publication year, sample size, research design, measurement tools, correlation coefficients, moderating variables (eg, gender ratio, cultural background). Since meta-analysis was used, the correlation coefficient r needed to be included. However, since some literature does not directly report the correlation coefficient r between lecturer job stress and occupational burnout, but only reports *F* value, t value, χ^2^ value or path coefficient *β*, we convert it into r value, that is, r = $\sqrt{\left[t2/(t2+\mathrm{df})\right]}$ , df = n₁ + n₂ − 2, r = $\sqrt{\left[F/(F+\mathrm{df})\right]}$ , df = n₁ + n₂ − 2, r = $\sqrt{\left[\chi 2/(\chi 2+N)\right]}$ , about the path coefficient β, if it is a multiple regression, a more general approximate formula can be used (Peterson and Brown, 2005): r = β + 0.05*λ*, λ = 1 (β is positive), λ = −1 (β is negative). If each analysis in the study involves only one predictor, then r ≈ β. If it is a multiple regression or there are covariates involved, r will be slightly smaller than β and cannot be directly equivalent, but it can be estimated by approximation.

### Quality assessment

In recent years, JBI has been widely used in the quality control stage of systematic reviews and meta-analyses (Hou et al., 2017), and is considered to be superior to the traditional NOS in the adaptability of cross-sectional studies (Xu et al., 2022; Barker et al., 2025). Since the 20 included studies were all cross-sectional studies, the JBI Critical Appraisal Checklist for Analytical Cross-Sectional Studies was used for quality assessment. Two reviewers independently scored each article according to the 8 criteria of the scale. Before the formal evaluation, the reviewers conducted trial scoring on some of the articles to ensure consistent understanding. Each item was scored according to “yes,” “no,” “unclear” or “not applicable.” In all studies, the two reviewers scored completely consistently, without the intervention of a third party. Given the complete consistency of the scores, consistency indicators (such as the Kappa coefficient) were not calculated. The final total score (0–8 points) was used to classify the literature into high quality (6–8 points), medium quality (3–5 points), and low quality (0–2 points) (Moola et al., 2017). Of the 20 studies included in this meta-analysis, 6 were rated as moderate quality by two independent reviewers, and 14 were rated as high quality. Overall, the quality of all included studies was moderate to high, providing a solid basis for this analysis.

### Statistical analysis (for meta-analysis)

All data analyses in this study were conducted using the Comprehensive Meta-Analysis software (Borenstein et al., 2021; Borenstein, 2022). The Pearson product–moment correlation coefficient (r) was chosen as the effect size. This approach helped to better examine the relationship between job stress and burnout among lecturers (Hunter and Schmidt, 2004). To interpret the strength of the correlations, the study followed Cohen’s (1992) guidelines. A small effect was defined as r = 0.10, a medium effect as r = 0.30, and a large effect as r = 0.50. The 95% confidence intervals were also calculated. Based on this information, the meta-analysis was then carried out. A random effects model was used. This decision was based on the expected variation among studies in terms of participants, settings, and measurement tools. The random effects model considers both within-study and between-study differences (Chen and Peace, 2013). Compared to the fixed effects model, it is more appropriate for combining research findings from diverse contexts. It also supports broader generalizations for future research.

This study used forest plots to show the effect sizes and confidence intervals for each included study. Funnel plots were also created to check for potential publication bias. To further assess publication bias, the Classic Fail-safe N method was applied. Statistical heterogeneity was examined using Cochran’s Q test and the I^2^ statistic. A significant *p*-value from the Q test (*p* < 0.05) suggested that heterogeneity was present. Based on the guidelines by Higgins et al. (2003), I^2^ values of 25, 50, and 75% indicated low, moderate, and high heterogeneity, respectively. To better understand the sources of heterogeneity, subgroup analyses and meta-regression were conducted. These analyses were based on pre-defined moderator variables, including sex ratio, cultural background, and the type of questionnaire used to measure the outcome variable.

## Results

### Study selection

**Figure 1:**
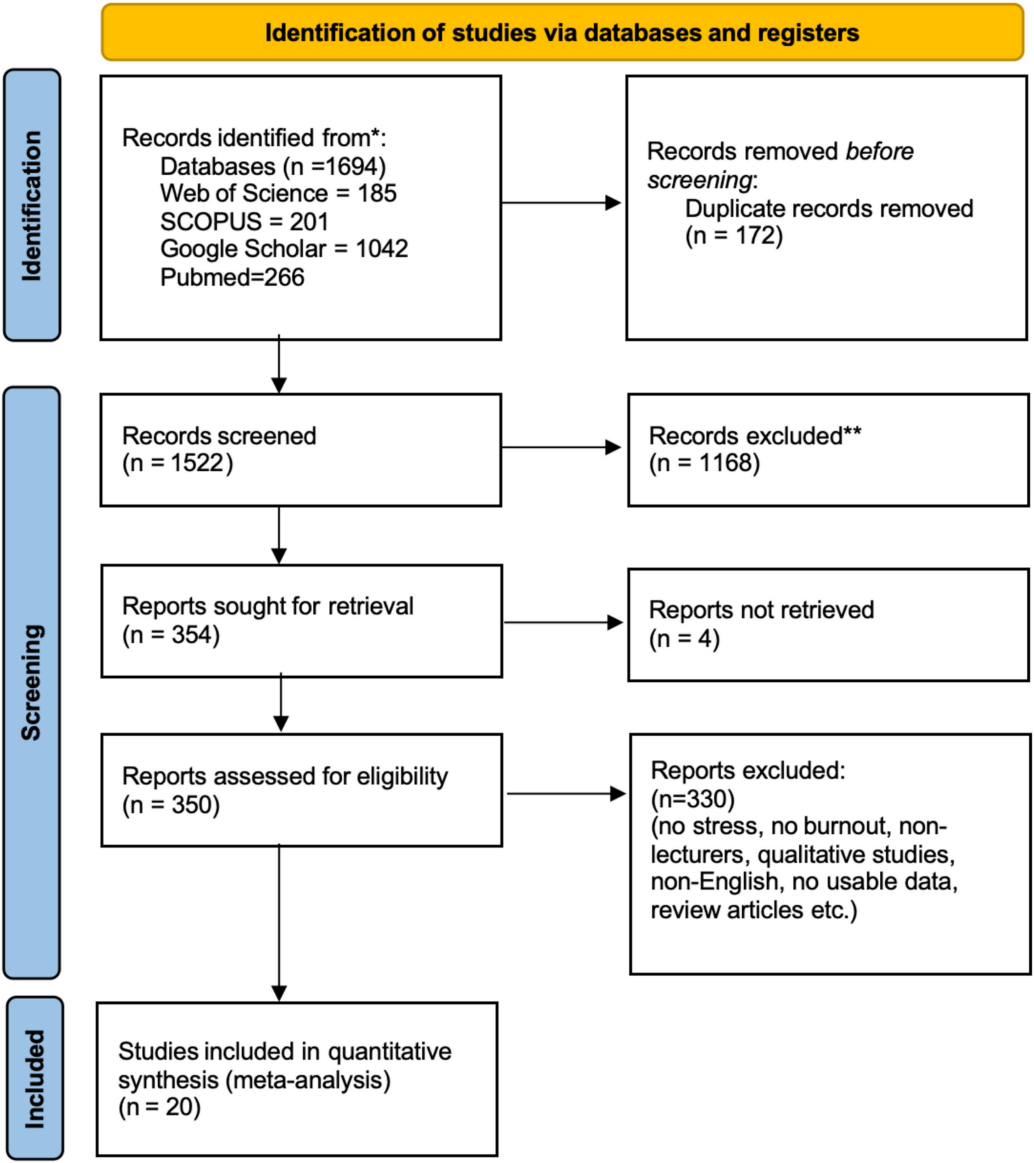
PRISMA flow diagram.

We conducted a systematic search in multiple databases using a Boolean search strategy and obtained a total of 1,694 records. Of these, 185 were from Web of Science, 201 from Scopus, 266 from PubMed, and 1,042 from Google Scholar. After preliminary processing, 172 duplicates were removed, and the remaining 1,522 articles entered the initial screening stage. A total of 1,168 articles were excluded through a rough screening of titles, keywords, and abstracts, and 4 articles were not available for full text. Therefore, 350 articles were left for rigorous and detailed screening. The next step involved a thorough screening process that continued to focus on titles, abstracts, and keywords. Among the retrieved articles, a total of 330 articles were excluded from the review due to a lack of keywords, non-English articles, review articles, and articles with a lack of extractable data. In the final stage, 20 articles were selected for detailed coding and analysis. The entire literature screening process can be presented in the PRISMA flow chart (Figure 1). Data management and screening were mainly completed using Microsoft Word and Excel, and references were collated and managed using Zotero. The full list and details of the 20 included studies are presented in Table 1. The measurement instruments used in these studies are summarized in Table 2.

**Table 1:** Details of included studies.

No.	Author(s)	Sample(N)	Stress measure	Burnout measure	Gender ratio	Country	Correlation(r)	Study type	Quality score
1	Abbas et al. (2012)	80	JSS	MBI-ES	blank	Pakistan	0.48 and 0.41	Cross-sectional study	5
2	Blix et al. (1994)	158	PWS	MBI-ES	2.854	California	0.61, 0.20, and –0.39	Cross-sectional study	6
3	Cao et al. (2024)	1,239	ASS	MBI	0.886	China	0.357	Cross-sectional study	5
4	Gomes et al. (2013)	333	SQAS	MBI-ES	0.665	Portugal	0.52	Cross-sectional study	7
5	Ishaq and Mahmood (2017)	240	DSS	MBI	1.124	Pakistan	0.137	Cross-sectional study	5
6	Jamal et al. (1998)	420	JSS	MBI	1.320	Canada	0.59	Cross-sectional study	6
7	Jiang et al. (2017)	1,092	UTOSS	MBI-GS	0.954	China	0.608	Cross-sectional study	7
8	Khan et al. (2018)	223	JSQ	OLBI	1.165	Pakistan	0.56 and 0.36	Cross-sectional study	6
9	Kim et al. (2025)	450	JSS	JBS	0.852	Thailand	0.57	Cross-sectional study	8
10	Li et al. (2022)	384	WSS	MBI-GS	1.201	China	0.776, 0.457, and 0.163	Cross-sectional study	6
11	Li (2018)	391	JSS	JBS	3.827	China	0.43	Cross-sectional study	5
12	Mahesar et al. (2020)	610	JSQ	MBI-ES	blank	Pakistan	0.745	Cross-sectional study	5
13	Norel and Necșoi (2017)	70	SPSA	MBI	0.029	Romania	0.371 and –0.273	Cross-sectional study	7
14	Pei et al. (2024)	7,565	SFSS	MBI-ES	0.613	China	0.600	Cross-sectional study	8
15	Rana and Soodan (2019)	412	OSI	MBI-HSS	1.497	India	0.525	Cross-sectional study	6
16	Salami (2011)	340	OSS	MBI-HSS	2.400	Nigeria	0.19, 0.25, and 0.20	Cross-sectional study	5
17	Teles et al. (2020)	520	PSS-14	MBI	1.873	Portugal	0.697, 0.420, and –0.365	Cross-sectional study	6
18	Wang et al. (2020)	1,906	SOSCT	JBUT	1.060	China	0.427	Cross-sectional study	8
19	Xu and Wang (2023)	202	RSS TSS ASS	EBS	0.629	China	0.57, 0.53, and 0.67	Cross-sectional study	8
20	Zhong et al. (2009)	300	OSI-2	MBI-GS	1.071	China	0.31	Cross-sectional study	7

**Table 2:** Questionnaires used for measuring burnout and stress in the studies included in the meta-analysis.

Abbreviation	Full name
Burnout	
JBUT	Scale of Job Burnout on University Teachers
MBI-HSS	Maslach Burnout Inventory-Human Services Survey
MBI	Maslach Burnout Inventory
MBI-GS	Maslach Burnout Inventory-General Survey
MBI-ES	Maslach Burnout Inventory-Educators Survey
OLBI	Oldenburg Burnout Inventory
BO	Burnout
EBS	Emotional Burnout Scale
Job stress	
SOSCT	Scale for Occupational Stressors on College Teachers
OSI	Occupational Stress Indicator
SPSA	Specific Professional Stressors in Academia
WSS	Job stress Scale
PWS	Perceived job stress
DSS	Daily Stress Scale
JSS	Job Stress Scale
OSS	Occupational Stress Scale
ASS	Academic Stress Scale
SFSS	Sources of Faculty Stress Scale
JS	Job Stress
PSS-14	Perceived Stress Scale
SQAS	Stress Questionnaire for Academic Staff
UTOSS	University Teacher Occupational Stress Scale
JSQ	Job Stress Questionnaire
OSI–2	Occupational Stress Indicator–2
RSS, TSS, and ASS	Research Stress Scale, Teaching Stress Scale, and Administrative Stress Scale

### Main meta-analysis

**Figure 2:**
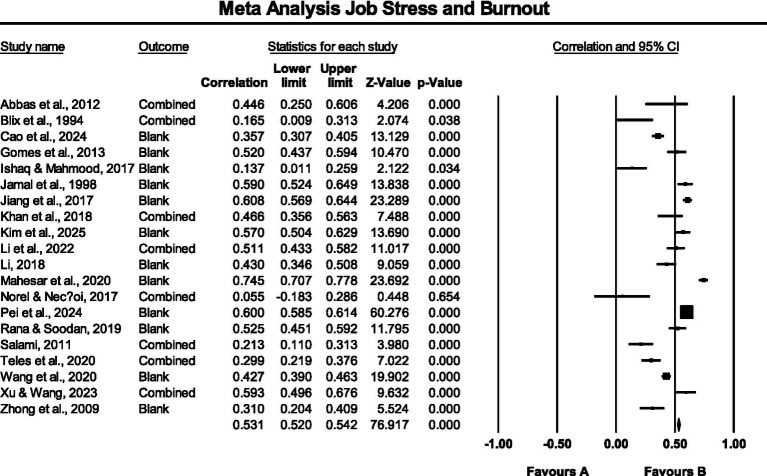
Meta-analysis forest plot.

The findings revealed that the combined effect size of the 20 studies was r = 0.452, the 95% confidence interval was [0.380, 0.519], Z = 10.911, *p* < 0.001, indicating that there was a significant positive correlation between the job stress and burnout. The Q value was 491.447, the degree of freedom df = 19, and *p* < 0.001, indicating that the variation of the effect size between different studies exceeded the range that could be explained by sampling error. The I^2^ value was 96.13%, suggesting that over 96% of the total variation among studies was due to actual differences rather than random error. This indicates a high level of heterogeneity. The Tau^2^ (τ^2^) value was 0.036, showing a considerable variance in effect sizes across studies. This result highlights the need to further investigate potential moderating variables or conduct subgroup analyses to better understand the sources of heterogeneity. The corresponding forest plot is presented in Figure 2.

### Sub-group analysis

Subgroup analysis and meta-regression analysis were used to explore heterogeneity. Cultural background and burnout measurement tools were important moderators affecting the effect size differences in this study, while gender ratio did not show a significant moderating effect in the current data. In terms of gender ratio, meta-regression analysis showed that its moderating effect was not significant (B = −0.0163, *p* = 0.748). In terms of cultural background, studies in Eastern cultural backgrounds reported higher effect sizes (r = 0.496, SE = 0.065, 95% CI = 0.420, 0.565) compared with Western cultural studies (r = 0.272, SE = 0.079, 95% CI = 0.117, 0.414), while the effect size in African cultural backgrounds was the highest (r = 0.590, SE = 0.048, 95% CI = 0.524, 0.649). The results showed that cultural differences may affect lecturers’ coping styles and the extent of their burnout experience when facing stress. For the burnout scale, studies using the MBI total scale reported a medium effect size (r = 0.315, SE = 0.084, 95% CI = 0.153, 0.461), while studies using the MBI-es scale reported a larger effect size (r = 0.529, SE = 0.088, 95% CI = 0.379, 0.652). Furthermore, the Burnout Scale (BS) reported an effect size of r = 0.570, which was higher than the other scales. In contrast, studies using the MBI-HSS reported a moderate effect size (r = 0.381, SE = 0.174, 95% CI = 0.042, 0.641), while studies using the OLBI reported a moderately high effect size (r = 0.466, SE = 0.056). This suggests that different measurement tools may differ in their sensitivity to assessing burnout, thus affecting the strength of the association.

### Publication bias

**Figure 3:**
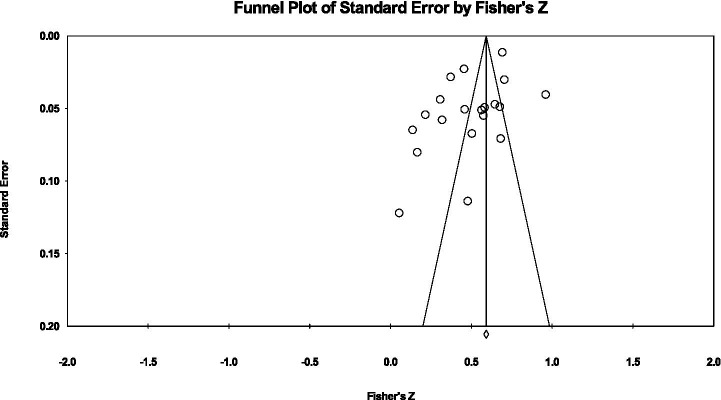
Funnel plot of standard error for burnout and job stress.

To assess whether the results of this study were subject to publication bias, this study used methods such as the classic fail-safe N, Orwin’s fail-safe N, as well as funnel plots and Egger regression tests for analysis. The classic fail-safe N results showed that 6,598 unpublished invalid studies (i.e., *p* value > 0.05) were required to make the overall effect of this study insignificant (Z = 56.50, *p* < 0.001). This value is much higher than the critical value recommended by Rosenthal (1979), that is, the fail-safe number should be >5 k + 10, indicating that the results of this study are highly robust. The Rosenthal method has limitations due to ignoring the systematic bias in unpublished studies, and may underestimate the actual risk of publication bias (Scargle, 1999). Therefore, Orwin’s fail-safe N was performed. When the average correlation coefficient of the observed study was r = 0.531 and the average correlation of the omitted study was assumed to be r = 0.00, a reasonable estimate could not be made (because the “insignificant” effect was also set to 0), suggesting that the setting parameters need to be adjusted for further supplementary analysis, but it is evident that the current effect size has a certain stability. In addition, the funnel plot is basically symmetrical and no obvious skew is observed (See Figure 3.). The results of the Egger regression test showed that the intercept term did not reach statistical significance (*p* > 0.05), further indicating that no significant publication bias was found in this study.

### Sensitivity analysis

We conducted a sensitivity analysis, assessing the impact of each study on the pooled effect size by excluding individual studies. The results showed that excluding any individual study did not significantly alter the pooled effect size (SupplemenmtaryTable S1), demonstrating the robustness of our findings. Furthermore, our analysis did not reveal any single study significantly contributing to the overall effect, further confirming the robustness of our conclusions.

## Discussion

In recent decades, the link between job stress and burnout has been a focal point of academic research. Among the 20 cross-sectional studies included in the meta-analysis, all studies showed a significant positive correlation. Numerous studies have found that job stress is most closely linked to emotional exhaustion, with moderate positive correlations to depersonalization and moderate negative correlations to personal accomplishment (Teles et al., 2020). At the same time, in a large number of studies, regression analysis results showed that job stress was a significant predictor of burnout (Salami, 2011; Abbas et al., 2012; Ishaq and Mahmood, 2017; Khan et al., 2018). Research results indicate that burnout is increasingly prevalent among faculty members facing workplace pressures such as mandatory, high-stakes performance evaluations (de Lourdes Machado-Taylor et al., 2016). Accumulated stress not only diminishes faculty well-being and job satisfaction but can also further impact their motivation and classroom performance (Hammoudi Halat et al., 2023). Furthermore, this stress-burnout synergy can lead to higher turnover rates and lower organizational performance, further impacting the overall academic atmosphere and institutional reputation (Zhang et al., 2023). Therefore, these findings suggest that universities should prioritize faculty mental health and optimize the organizational environment. By alleviating occupational stress through appropriate workload distribution, psychological support services, and fostering a positive organizational culture, they can reduce the risk of burnout and enhance overall institutional effectiveness.

In addition, this study used subgroup analysis and meta-regression analysis, finding that cultural background and burnout measurement tools were important moderators of the effect size differences in this study. Regarding cultural background, the impact of job stress on job burnout among lecturers in Asian and African cultural backgrounds was greater than that in Western cultures. In other words, lecturers in Asian and African countries may be more sensitive to burnout when faced with job stress. These differences may be closely related to cultural background, institutional environment, and policy factors. Cultural attitudes may contribute to different perceptions of working hours. In particular, in collectivist cultures, employees in Asia and Africa tend to work longer hours and experience heavier workloads, leading to higher levels of job stress. This is because collectivist cultures emphasize social harmony, a strong sense of family and social responsibility, and respect for authority, and these cultural values often translate into a high acceptance of long working hours (Abdul Aziz and Ong, 2024). Meanwhile, a study found that employees who work 40–50 h or more per week (the average weekly working hours in the region) are more likely to experience burnout (Abdul Aziz and Ong, 2024). In addition, lack of effective social support is another key factor that exacerbates the job stress of lecturers in Asia and Africa. In many Asian and African countries, lecturers lack adequate career support systems and resources, which are manifested in limited professional development channels, scarce promotion opportunities, a lack of scientific research funding, and a shortage of basic teaching facilities and equipment. In China, lecturers often face a demanding teaching schedule, typically working 40 to 50 h per week. Lecturers not only shoulder extensive teaching responsibilities but also need to participate in research and administrative work. However, these additional tasks often lack support and resources, leading to physical and mental exhaustion (Sun et al., 2011; Sun et al., 2023). Lecturers in South Africa face a similar situation, working 40 h a week, which exacerbates workload and stress, particularly in a resource-constrained environment. Many educators are juggling teaching, research, and materials development, leaving little time for rest or mental well-being (Razalli et al., 2021; Aboagye Akuffo et al., 2023). It can further exacerbate burnout, which is caused by the difficulty in getting timely help and mental health support when facing job stress. Given that different cultural backgrounds may have different influences, future research should pay more attention to the diversity of cultural backgrounds, so as to gain a more comprehensive understanding of their impact on job stress and burnout.

Regarding burnout scales, studies using the MBI-ES and Burnout Scale (BS) reported higher effect sizes (r = 0.529 and r = 0.570), while studies using the MBI Total Scale and MBI-HSS reported lower effect sizes (r = 0.315 and r = 0.381). The effect size of the OLBI scale was intermediate between the two (r = 0.466). The influence of different scales on effect sizes may primarily be due to their design focus and sensitivity to various dimensions of burnout. Among these, the most widely used and commonly employed burnout questionnaire is the Maslach Burnout Inventory (MBI) (Schaufeli et al., 2001). It was originally designed to assess burnout among workers in the human services sector (MBI-Human Services Survey, MBI-HSS). As research deepened, the scale was gradually revised and expanded to encompass a wider range of occupations, resulting in versions such as the MBI-General Survey (for non-human services occupations) and the MBI-Educators Survey (for educational institutions) (Halbesleben and Buckley, 2004). However, some researchers believe that the personal achievement dimension should be excluded from the MBI (Demerouti et al., 2001). Abbas et al. (2012) found that sensitivity to personal achievement was low, especially in certain industries or occupations, and that this dimension lacked correlation with stress, suggesting that this dimension may be independent of the stress-burnout path. Moreover, some researchers have argued for the use of a simpler, more generalizable measure. Schaufeli and Bakker (2004) evaluated the OLBI and found it to be more sensitive than the MBI in assessing exhaustion and disengagement, particularly in occupations with higher workloads and less-complete measures of emotional exhaustion. The Oldenburg Burnout Inventory (BVI) includes the dimensions of exhaustion and disengagement. Exhaustion encompasses not only mental fatigue but also physical fatigue, a strong need for recovery, and the effects of excessive workload. Disengagement, on the other hand, describes a feeling of detachment from work tasks and responsibilities and can be used as an alternative to the MBI-GS scale (Korunka et al., 2010). In summary, scales focused on emotional exhaustion (such as the MBI-ES) typically report high effect sizes because they accurately measure core indicators of burnout. More comprehensive scales (such as the Burnout Scale and the OLBI) capture all dimensions of burnout more comprehensively and therefore typically exhibit high or moderately high effect sizes. In contrast, scales like the MBI Total Scale and the MBI-HSS have lower effect sizes, perhaps due to their generality or reduced sensitivity when applied in specific occupational contexts. This impacts the relationship between lecturer job stress and burnout, and future research needs to select more appropriate measurement tools.

The present review highlights the various mediating variables. Simultaneously, burnout is not only the result of job stress, but may also affect other work-related variables as a mediating variable. Burnout is a key mediating variable affecting teachers’ job satisfaction and turnover intention (Mahesar et al., 2020). For example, burnout plays a stronger role as a mediating variable between job stress and turnover intention than job satisfaction, indicating that teachers first experience emotional exhaustion and decreased motivation under high stress, which in turn affects their willingness to stay (Wang et al., 2020). Burnout is associated with work engagement and serves as a crucial mediator between stress and engagement. In addition, it also indirectly affects job satisfaction by reducing job engagement (Li, 2018). Although stress has no direct predictive power on job satisfaction, it has a significant indirect effect through the mediating effect of burnout (Kim et al., 2025). In the job stress-burnout path, a large amount of research indicates that self-efficacy has a significant moderating effect on the relationship, indicating that people with high self-efficacy are more likely to avoid burnout tendencies when facing stress (Ishaq and Mahmood, 2017; Xu and Wang, 2023). Occupational burnout has a medical definition, especially some studies have discovered that occupational burnout plays a mediating role between job stress and physical and mental health (such as depression and physical discomfort) (Zhong et al., 2009; Jiang et al., 2017). This finding seems to be consistent with the review of Koutsimani et al. (2019). Because the researchers tried to distinguish the overlapping concepts of burnout, depression and anxiety in the review, this study also clarified the overlapping parts of burnout and job stress while exploring the relationship.

Conversely, this study provides empirical evidence for the framework of theory. Cao et al. (2024) verified the applicability of the job demand-resource model and equity theory in explaining the mechanism of occupational burnout of college teachers. Blix et al. (1994) and Salami (2011) both emphasized the importance of matching the work environment with teachers’ personal motivation in their studies, supporting the applicability of the P-E Fit theory. Pei et al. (2024) and Khan et al. (2018) believed, based on the conservation of resources theory (COR), that if individuals cannot obtain sufficient resources to cope with stress, stress accumulation will occur and lead to burnout. At the same time, the results of this study are consistent with the “Job Demand-Resource Model” (JD-R) and Maslach’s burnout theory, which believe that high job demands are more likely to lead to emotional exhaustion and burnout in a situation of insufficient resources (Cao et al., 2024).

Research indicates that teacher burnout is related to personal factors and work factors. In the literature, there are differences in the strength of paths between different types of colleges and universities (Wang et al., 2020). Studies have also pointed out that the stress level of teachers in private colleges and universities is significantly higher than that in public colleges and universities, indicating that organizational support and individual psychological resources jointly affect the occupational health status of teachers (Ishaq and Mahmood, 2017). Moreover, gender and age significantly influence the mechanisms underlying stress and burnout among college educators. Female teachers generally feel higher stress, especially in terms of work–family conflict and interpersonal interaction, and show higher levels of emotional exhaustion (Norel and Necșoi, 2017). The difference between university lecturers and other teachers is the heavy teaching and research tasks (Rana and Soodan, 2019).

This study systematically reviewed and comprehensively summarized the relevant research. The summary effect size of the correlation was derived from a meta-analysis. Systematic literature reviews have great limitations and may influence the scope and detail of the results, but meta-analysis can provide sufficient evidence to support relevant interventions or other research phenomena. It is significant as it presents a thorough overview of the concepts and measurement instruments, while also offering statistical data that supports the correlation and influencing factors involved. This research provides important insights into the relationship between job stress and job burnout among university lecturers.

## Study limitations

This study systematically reviewed and synthesized the existing literature, but it still has several limitations. First, meta-analysis relies on existing data for inclusion, and studies lacking relevant data may have been excluded. Language and search limitations may also have led to omissions of some studies. Second, although we searched and screened a large number of studies, the vast majority of studies that met the inclusion criteria employed cross-sectional designs, which can only reveal correlations between variables but cannot establish causal relationships. Future research could focus more on longitudinal and experimental designs. Third, the sample size included in this study was primarily concentrated in Asian countries, particularly China and Pakistan. This geographic concentration may limit the applicability of the findings to other cultural contexts and higher education systems. Future research should expand to more diverse regions, such as Europe, North America, and Latin America, to enhance the generalizability of the findings. Numerous instruments exist for measuring burnout. This study only selected scales used in the included studies for moderated analysis. Furthermore, the burnout measurement instruments used in the included studies varied, and this study was limited to the scales used in the original studies, which may increase heterogeneity in the results. Finally, this study only examined the moderating effects of cultural background, measurement tools, and gender, and did not consider potential variables such as academic level, teaching load, work-life balance, or contract type. Future research could further explore these factors to expand and deepen this study’s findings. In summary, future research should continue to expand in terms of research design, geographical distribution, and the diversity of moderating variables to enhance the robustness and cross-cultural applicability of the conclusions.

## Practical implications

Through systematic literature review and meta-analysis, this study found that the degree of lecturers’ job burnout is usually related to the degree of job stress. In short, the higher the lecturer’s job stress level, the more severe the burnout, and burnout has a significant negative impact on teachers’ physical and mental health, job satisfaction and educational quality. This finding not only provides important insights for higher education institutions but also aligns closely with global initiatives to improve teacher well-being and the sustainability of academic environments. Meanwhile, UNESCO has proposed several initiatives in its Global Teacher Report aimed at improving teachers’ working conditions and professional development, thereby ensuring the stability and sustainability of education systems (UNESCO and Education 2030 International Task Force, 2024).

The World Health Organization (WHO) recommended in its 2022 Global Guidelines for Mental Health in the Workplace that measures be taken to address mental health risks, such as heavy workloads, negative behaviors, and other sources of job stress. The guidelines propose that managers should be trained to proactively prevent the creation of high-pressure work environments and provide effective support to employees in difficult situations. In response to lecturers’ job stress and burnout, universities should adopt a series of effective intervention measures to optimize the working environment of faculty and staff. Specific measures include: First, reasonable workload distribution is crucial. Through scientific and reasonable workload adjustments, teachers’ excessive burdens can be reduced, helping them to better balance teaching, scientific research, and administrative work. Second, psychological counseling and support services should become regular services in higher education institutions, regularly providing mental health support to teachers to help them cope with job stress. Furthermore, providing more research support and resource investment can alleviate the pressure on teachers in scientific research and enhance their academic development and career satisfaction. Finally, establishing a mentor system means that helping young teachers enhance their professional identity and sense of accomplishment through the guidance of senior teachers, thereby effectively reducing the risk of burnout.

Therefore, this study provides empirical support for further research on faculty well-being and mental health interventions. The findings are not only valuable for promoting work-life balance among higher education faculty, but also highlight the central role of faculty well-being in sustainable academic development. Based on above, we recommend that educational institutions actively respond to global initiatives and systematically implement interventions to ensure the long-term stability and development of their academic workforce, thereby improving faculty mental health, promoting the overall progress of educational institutions, and enhancing their international competitiveness.

## Conclusion

Although numerous studies have confirmed the correlation between job stress and burnout, there is still a lack of systematic literature reviews and meta-analyses for higher education lecturers. Therefore, this study conducted a systematic literature review supplemented by a meta-analysis. The results found a significant positive correlation between job stress and burnout among higher education lecturers. This result is consistent with several existing studies included in the study, which also shows that the greater the lecturer’s job stress, the higher level of burnout, and this stress also affects other work-related variables, such as job satisfaction and intention to leave. Based on the results of this study, it is recommended that universities should take effective intervention measures as soon as possible to reduce lecturers’ job stress and burnout, thereby promoting lecturers’ mental health and overall well-being.

## Data Availability

The original contributions presented in the study are included in the article/supplementary material, further inquiries can be directed to the corresponding author.
